# Scale, context, and heterogeneity: the complexity of the social space

**DOI:** 10.1038/s41598-022-12871-5

**Published:** 2022-05-31

**Authors:** José Balsa-Barreiro, Mónica Menendez, Alfredo J. Morales

**Affiliations:** 1 MIT Media Lab, Massachusetts Institute of Technology, 75 Amherst Street, Cambridge, MA 02139 USA; 2 Division of Engineering, New York University Abu Dhabi, Saadiyat Island, P.O. Box 129188, Abu Dhabi, United Arab Emirates; 3 New England Complex Systems Institute, 277 Broadway, Cambridge, MA 02143 USA

**Keywords:** Socioeconomic scenarios, Environmental economics

## Abstract

The *social space* refers to physical or virtual places where people interact with one another. It decisively influences the emergence of human behaviors. However, little is known about the nature and complexity of the social space, nor its relationship to context and spatial scale. Recently, the science of complex systems has bridged between fields of knowledge to provide quantitative responses to fundamental sociological questions. In this paper, we analyze the shifting behavior of social space in terms of human interactions and wealth distribution across multiple scales using fine-grained data collected from both official (US Census Bureau) and unofficial data sources (social media). We use these data to unveil how patterns strongly depend upon the observation scale. Therefore, it is crucial for any analysis to be framed within the appropriate context to avoid biased results and/or misleading conclusions. Biased data analysis may lead to the adoption of fragile and poor decisions. Including context and a proper understanding of the spatial scale are essential nowadays, especially with the pervasive role of data-driven tools in decision-making processes.

## Introduction

Since the onset of the COVID-19 pandemic in early 2020, the world has entered a negative spiral of instability and uncertainty. The health emergency has been followed by socio-economic and political crises and conflicts throughout the globe, with an uneven distribution of impacts across different regions. In general, governmental authorities and the bulk of the population still seem overwhelmed, with many of them having difficulties coping with the continuous flow of events. Undoubtedly, all these events show the complexity of human societies nowadays. Therefore, we first must improve our understanding of this complexity in order to adopt an optimal decision-making process.

Human communication is crucial for building trust, establishing cooperation, and achieving collective action in social systems^1^. The *social space* refers to all those places where people meet and interact with each other, be it physical or virtual. This space is defined within a particular spatial and temporal context. It is influenced by the information generated by people involved in it, but also by the information coming from other (neighboring) social spaces. Collective behaviors in human societies depend on the underlying social system, but also the context where they emerge. They might be shaped by large-scale features such as social norms and cultural customs (ethnicity, nationality, or religion), but also particular preferences related to the sense of belonging to a community. The complexity behind social spaces can be embraced by considering both the topology of social networks and the multiscale facets of collective dynamics. Concerning the first issue, Balsa-Barreiro et al.^2^ demonstrated how societies develop the right conditions for their own collapse. They modeled the structure of socio-economic interdependencies by demonstrating that an excessive number of interconnections was associated with a higher risk of systemic fragility due to the emergence of *cascading effects*. Before the emergence of COVID-19, this study warned about the limitations of excessive globalization and the consequent risk of collapse. However, changes in the properties of social systems across multiple scales are not well understood yet.

In the past, research on *social space* was spatially limited to certain spatial scales based on reduced sample sizes. However, social systems often develop different behavioral patterns depending on the scale. To address this concern, geographers identified the *modifiable areal unit problem* (MAUP), one of the most longstanding and far-reaching problems in geography. MAUP is a kind of statistical bias that can significantly influence the results of statistical hypothesis tests. In essence, the MAUP refers to the sensitivity (or inconsistency) in geospatial analysis once we use different spatial configurations ^3,4^. MAUP can be further divided into two different sub-problems, i.e. (a) the spatial scale and (b) the zoning sub-problem^5^. The first one refers to the sensitivity of results based on the aggregation level of data observations and, in consequence, the number of spatial units used. The second one refers to the effect of moving or redrawing the boundaries of spatial units, but always keeping invariant the same number of spatial units^6^. In other words, geospatial analysis can substantially vary their outcomes depending on both *how* and *how much* we aggregate our data. This is even more relevant by knowing that boundaries of many geographical units are often demarcated artificially and these could be redrawn, which could lead to inconsistent results^5^. Carballada and Balsa-Barreiro^7^ discussed the influence of the MAUP effect in mapping fine-grained COVID-19 data, showing variability in the results and other concerns such as revealing private identities in some spatial contexts. MAUP is an ever-recurrent problem in human dynamics research^8,9^ such as spatial distribution of migrations^10^, residential segregation^11^, or criminality^12^, among others.

The impact of the spatial scale is also shown in *fractal dimensions*. Their basic structure is fragmented and apparently irregular, which tends to be repetitive across multiple scales. Fractals refer to complex patterns that emerge repeatedly in an ongoing feedback loop according to the *self-similarity* principle. In his renowned study, Mandelbrot^13^ attempted to measure the length of the British coast. He realized that the measured length of a coastline’s stretch depends on the scale of measurement, showing his early thinking on fractals. The so-called *coastline paradox* is the counterintuitive observation that the coastline does not have a well-defined length. Batty and Longley^14^ demonstrated the emergence of fractal patterns revealing the complexity and diversity of the apparently irregular urban sprawl processes across the globe. Balsa-Barreiro et al.^15^ demonstrated how urban dynamics that are uniquely expected in cities, emerge paradoxically in eminently rural areas with population decline, where a much-reduced number of human settlements tend to concentrate most of the population over time.

On the other hand, the traditional partition between disciplines in social sciences shows certain limitations for a complete understanding of the complexity of the social space. Both sociologists and geographers have applied a static vision of space where the complex interactions between social groups are often under-represented. Further, most of these studies rely on small sample sizes. In consequence, the complexity of social space across multiple scales is partially misunderstood. The science of complex systems bridges between disciplines for finding out the existence of deterministic mechanisms behind collective behaviors^16^. To that end, they apply a number of concepts from physics, including interaction networks, chaotic dynamics, extreme event theory, pervasive uncertainty, and contextual conditioning^17–19^.

Nowadays, the explosion of *big data* from individual mobile phones has led to new approaches for understanding much better collective behaviors of the underlying social systems. Data collected from individual interactions in social media together with other sources (such as US Census Bureau data) allows to identify patterns of collective behaviors within their underlying social structures and spatial contexts^20^. In fact, some recent studies have leveraged these new data opportunities to address the complexity of social dynamics. For instance, a successive group of studies demonstrated how anonymous location data of mobile phone users delivers important insights into the mobility patterns of individuals and communities^21^. Gonzalez et al.^22^ analyzed the trajectory of 100,000 anonymized mobile phone users whose position was tracked for a six-month period. They demonstrated the temporal and spatial regularity of human trajectories, with each person exhibiting a time-independent travel distance and a high likelihood of returning to a few highly frequented locations. Song et al. ^23^ explored the limits of predictability in human dynamics by studying the mobility patterns of anonymized mobile phone users. Despite significant differences in travel patterns, they found a 93% potential predictability in user mobility across the whole user baseline. This leads to reproducible traffic patterns at an aggregate level for any given city even over periods of 1 year^24^. Lu et al. ^25^ concluded that human mobility is highly dependent on historical behaviors. A similar conclusion was reached by Loder et al. ^26^, which analyzed billions of vehicle observations in 41 cities across the globe. They demonstrated that network topology determines traffic capacity, making traffic congestion somewhat predictable. Shi et al. ^27^ analyzed data collected from 1 million users in Harbin, a large city in northeastern China, over one month. They found that people tend to communicate with individuals nearby, and suggested that the distance decay effect along with the home-to-work distance contribute to several distribution patterns of social communities. Dmowska and Stepinski^28,29^ analyzed racial diversity across United States for the period 1990–2010. In the case of urban environments, they analyzed spatial patterns of residential racial segregation in 41 American cities drawing distributions that are either power-laws or approximate power-laws. Dong et al. ^30^ analyzed how income segregation determines social interactions both offline and online. They checked preferred discussion topics in the online space according to income in some European and American cities. Discussions in wealthy neighborhoods typically included life-style topics such as traveling or leisure, while in poor neighborhoods discussions focused on sports or TV shows.

In this paper, we demonstrate the relevance of spatial scale in the analysis of the social space. To this end, we study the shifting behavior of social space across multiple scales by analyzing fine-grained data from several sources (social media and US Census Bureau) related to human interactions and wealth distribution. Our approach provides a framework for a holistic and more comprehensive understanding of human dynamics and social systems. The rest of this paper is organized as follows: “Data” introduces the datasets. “Methodology” describes briefly the methodology used for data processing and visualization. “Results” presents the results across multiple scales. “Discussion and conclusions” discusses the relevance of results.

## Data

We collect data from two data sources: Twitter and the US Census Bureau. Twitter data was collected by querying Twitter’s Stream API. We gather over 70 million tweets between August and September 2019, which were posted by 4 million users across the globe. Collected tweets were geotagged with the spatial coordinates from where they were posted. The geolocation of tweets indicates where the users are located, but it is also possible to infer spatial interactions between users via mentions or retweets.

Data collected from the US Census Bureau^31^ refers to income distribution in the United States. These data are aggregated at the census tract level for 2017. We map historical income composition over time by collecting income data for the period 1969–2017. In the case of historical income data, these are aggregated at the county level. The United States counts with 73,057 census tracts and 3143 counties. Hence, our database includes more than 100,000 records. Administrative entities are represented by nodes, which are progressively aggregated based on distance and spatial distribution criteria. In this research, we extract spatial patterns related to income composition across the United States based on the mesh size used for data aggregation.

## Methodology

For the Twitter data analysis, we implement spatial networks following the methods described by Hedayatifar et al. ^32,33^. Firstly, we geolocate all tweets based on their coordinates. Secondly, we create a mesh over the geographical map and then define the size of each location based on the latitude and longitude coordinates of their corners. Nodes represent individual places from where tweets were sent, and links show spatial interactions between users. In the case of online communication networks, links are created when user *u* at location *i* mentions another user *b* at location *j*. In the case of mobility networks, links emerge when user *u* tweets first from location *i* and then from location *j*. Links are weighted by the number of people who either travel or communicate between *i* and *j*. Nodes and links allow to unveil the structure of social networks across multiple scales and geographical contexts. Once the networks are created, we analyze their structure using #NetworkX and #SciPy libraries in Python. We use GIS tools such ArcGIS Desktop and Mapbox for mapping spatial networks.

Spatial networks obtained from Twitter data are simplified into partitions or communities. After that, we cluster the networks by applying the *Louvain algorithm*, one of the most popular methods for community detection^34^. In this way, spatial networks are fragmented into non-overlapping communities where the nodes within are reciprocally connected. The *Louvain algorithm* finds the optimal value of modularity to ensure the quality of the partition. This algorithm is iteratively applied on the dataset in order to identify the most reliable fragmentation of the social space into communities.

In the case of the US census data, we characterize census tracts and counties by the geographic coordinates of their centroids. The distances between centroids are estimated using #ScikitLearn in Python. This library has a function named *nearest neighbors* that returns the number of entities within a given radius around each centroid. The multiscale analysis is performed by varying the radius distance. Maps are implemented in #Matplotlib and #Cartopy in Python, which facilitate querying and displaying geographic information.

## Results

Results are organized in two sub-sections: (4.1) Community detection from the Twitter dataset, and (4.2) Spatial patterns based on income composition data extracted from the US Census Bureau dataset.

### Community detection

**Figure 1:**
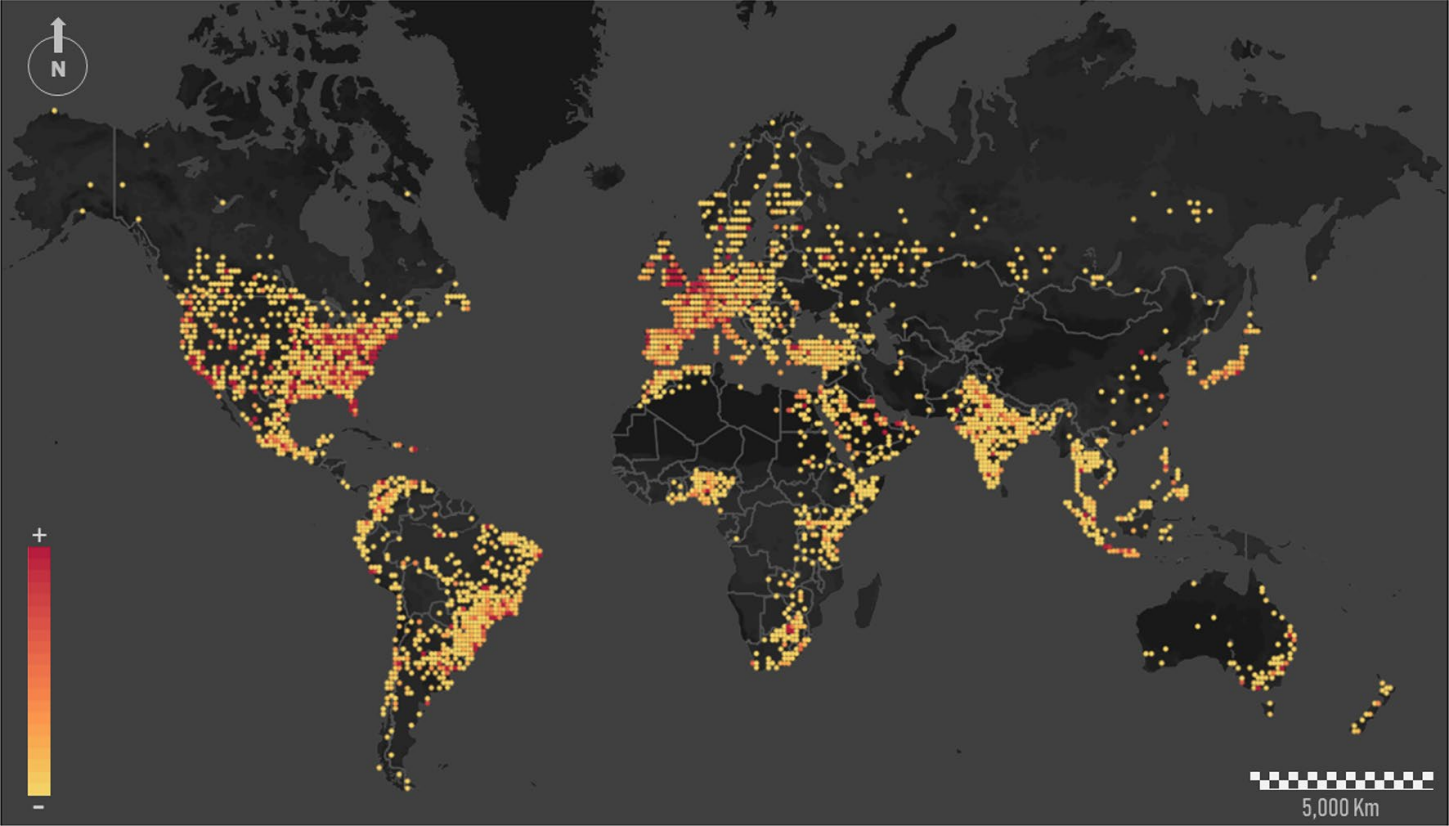
Global heatmap showing tweets location. Main hotspots are shown in red. This figure was generated using ArcGIS Desktop and Mapbox.

The extraction of global communication patterns can be inferred from Twitter. Figure 1 shows Twitter activity on a global scale. Nodes correspond to the physical location where tweets were sent. These nodes are displayed in a heatmap where yellow to red regions concentrate more activity. The map obviously shows certain similarity with a global population map, where the most densely populated regions are highlighted. However, some particularities are evident. China, India, and other countries located in southeastern Asia show much lower node densities in comparison to their actual demographic weight. One potential explanation is because the accessibility to the Internet is restricted for people with limited resources, but also because they have their own national microblogging services and social networks. In the case of the African continent, its actual demographic weight is clearly underestimated.

**Figure 2:**
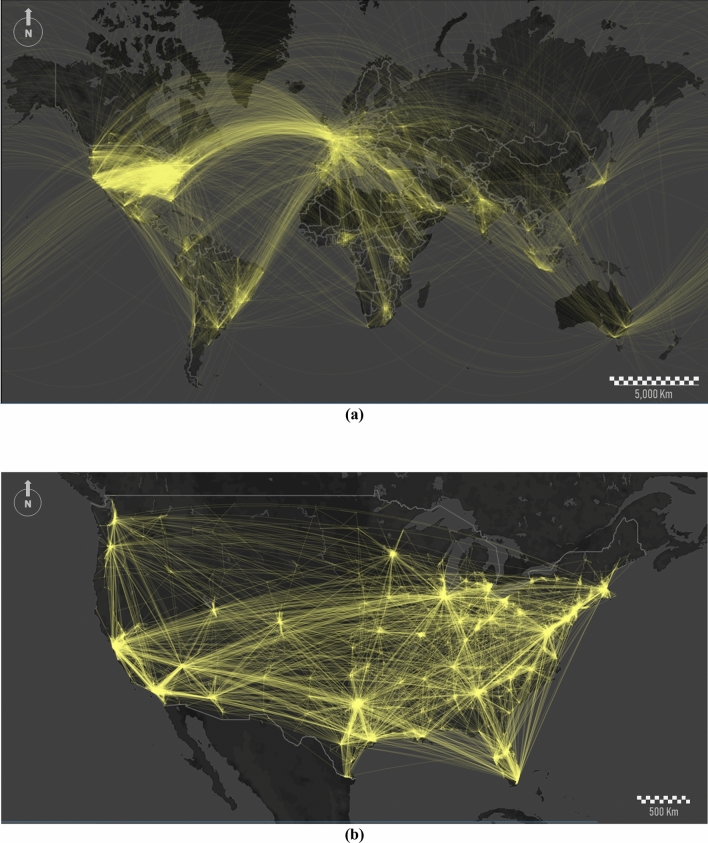
Flows of interconnectivity between users via mentions or retweets: (**a**) global scale, and (**b**) the United States mainland. To make the display clearer, we apply a thinning algorithm for reducing line densities. We reduce the total number of links by 90% in (**a**) and 95% in (**b**). This figure was generated using ArcGIS Desktop and Mapbox.

The interconnectivity between nodes is estimated based on mentions or retweets. It is represented by flows (Fig. 2a). The spatial networks show different configurations based on the regions where tweets come from and go to. Thus, a very dense global network emerges between the United States, Europe, and some other particular hotspots. Additionally, we observe other densely connected local networks in small regions such as Japan, East Asia, India, South Africa, and some particular regions in Latin America. Zooming into the United States mainland (Fig. 2b), the interconnectivity between both coastline sectors and the entire eastern half of the country becomes more evident.

These maps reflect the spatial heterogeneity of global human dynamics. The traditional dominance of some western countries is also reflected in Twitter. Nodes summarize regions where the wealth is concentrated. Thus, the global network’s topology is a proxy for dominant mobility flows traced by migrations, commercial relationships, and preferred trade routes across the globe. This interconnectivity shows aspects related to cultural dominance, where the majority of the western countries share the same entertainment industry (and knowledge of a common language). Local networks represent regions with enough cultural, geopolitical, or economical affinity for ruling communication within their influence areas.

The collective identity is the common structure of beliefs, values, symbols, and behaviors that result from our association in communities. Axelrod^35^ argues that the collective behavior is mostly determined by an evolving and complex process of human interactions and information accumulation over time. We learn by imitation and therefore, we are prone to become similar to those that we are exposed to and frequently interact with. Initial differences between communities’ behaviors are reinforced over time, which leads to their eventual divergence and the emergence of multiple cultures.

Fragmentation and clustering of the social space allows to detect communities where people preferably interact with each other by defining the way that trade routes are predominant. Hedayatifar et al.^32^ found a significant correlation between the level of communication and the topology shown by international trade networks. Geographical distances and neighborhood relationships are two relevant factors^36^, but not the only ones. Historical past, geopolitical relationships, and cultural influence between countries are equally important for understanding the map of global interactions on Twitter.

**Figure 3:**
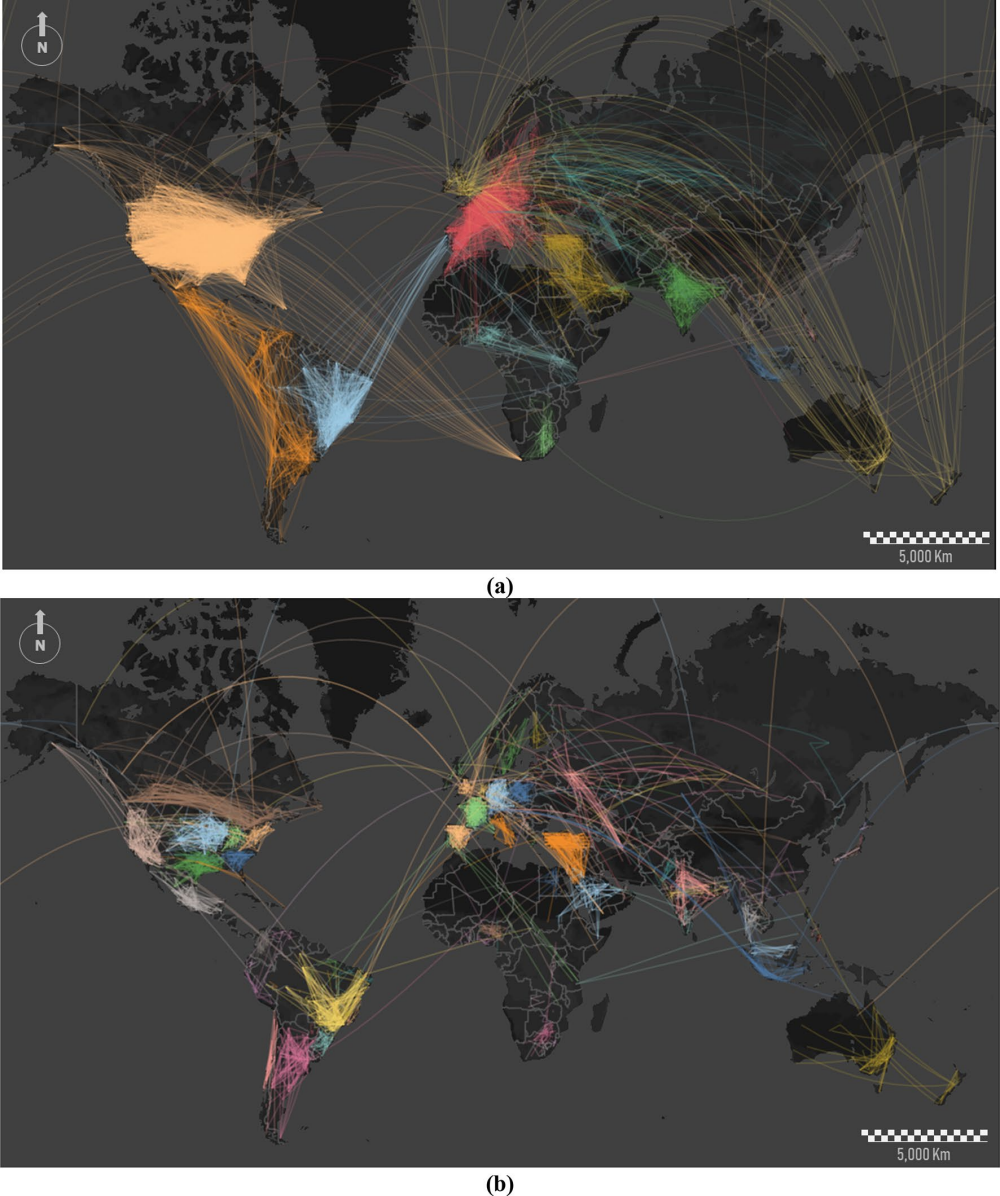
Node clustering and detection of communities/sub-communities at a global scale from Twitter dataset. We detect (**a**) 14 major communities and (**b**) 86 sub-communities. Consistent partitions were obtained over 85% of realizations. To make the display clearer, we apply a thinning algorithm for reducing line densities. We reduce the total number of links by 90%. This figure was generated using ArcGIS Desktop and Mapbox.

For community detection, we map the Twitter dataset into a lattice composed of a regular grid with 100 km wide cells. We then run the *Louvain algorithm* to partition the whole network into regions and to identify the clusters with the highest interconnectivities. On a global scale, we identify 14 major communities (Fig. 3a) and 86 minor communities or sub-communities from the subsequent fragmentation of the first ones (Fig. 3b). Clusters imply specific cross-cultural, cross-national, and/or cross-linguistic associations. In the Americas, three large communities are differentiated due to language. But once we run the algorithm in successive iterations, minor communities emerge in Latin America. It is noticeable that throughout the fragmentation process some of the clusters are equivalent to nations (Brazil, India, and several European countries), whereas in the case of the United States, it is internally partitioned into different sub-communities within, showing a rich cultural diversity.

**Figure 4:**
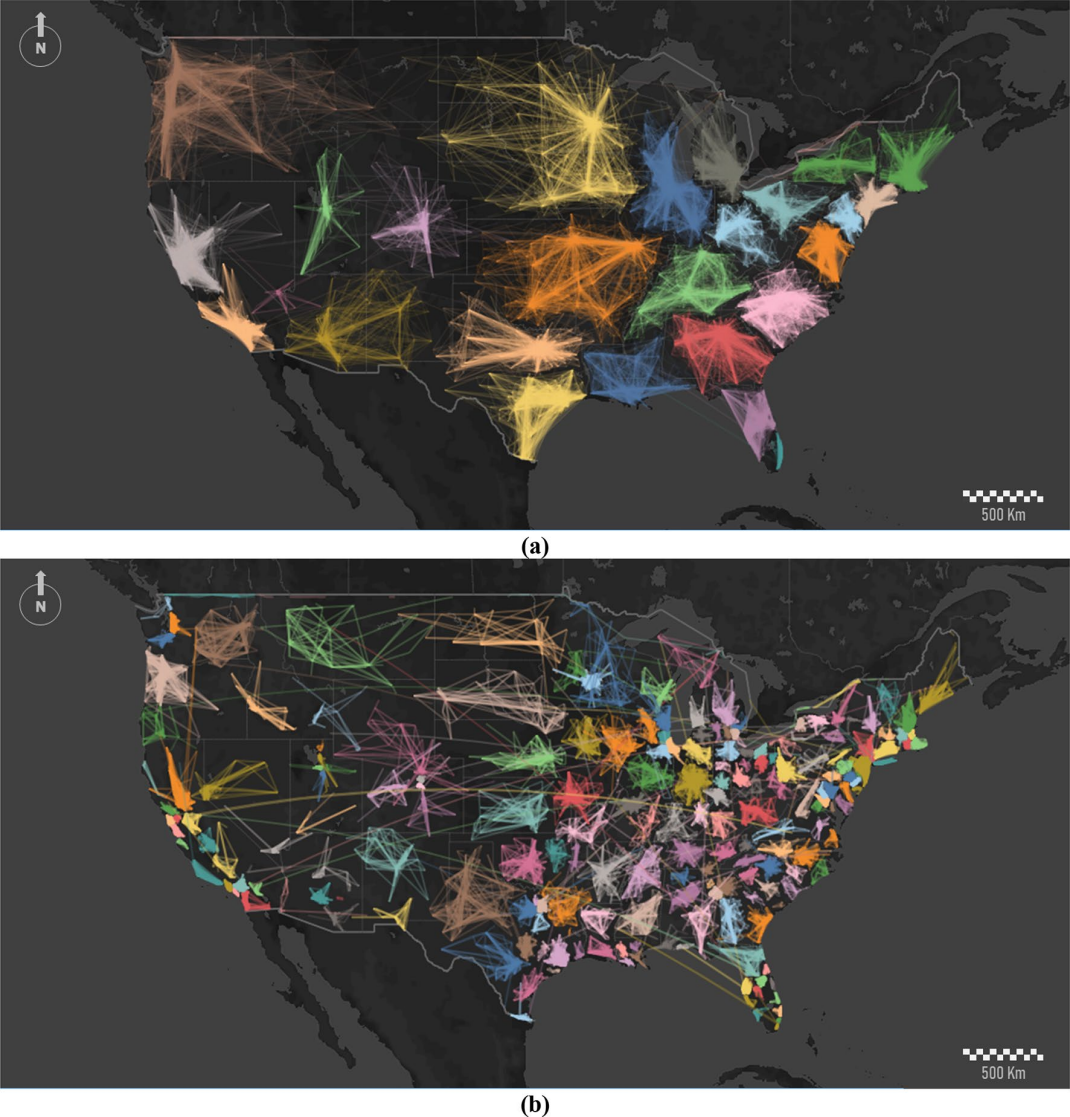
Node clustering and detection of communities/sub-communities from Twitter dataset in the United States mainland. We detect (**a**) 39 major communities and (**b**) over 216 sub-communities. Consistent partitions were obtained over 85% of realizations. To make the display clearer, we apply a thinning algorithm for reducing line densities. We reduce the total number of links by 40%. This figure was generated using ArcGIS Desktop and Mapbox.

Further zooming into the dataset and running the algorithm for a particular region allows us to refine the results. In Fig. 4, we show communities and sub-communities across the mainland United States. In this case, we overlay a grid of 10 km wide cells (see Hedayatifar et al.^33^). As it is evident, the internal clustering differs according to the scale, showing relevant changes in both the number and size of the partitions. The reduced number of communities detected corresponds mostly to vast regions surrounding the most populated cities in the US central states (Fig. 4a). Most communities are far more extensive than their own states, which is obvious as the number of communities is lower than the number of states. This effect is particularly clear with the integration of North and South Carolina into one single cluster, but also in New England. On the other hand, the state of California is internally partitioned into two different communities due to the influence of San Francisco in the north and Los Angeles in the south. At this scale, the number of sub-communities increases substantially up to 216, as shown in Fig. 4b. Again, some states show a clear homogeneity with a unique dominant cluster (Maine, Montana, and Wyoming), whereas the great majority show a clear diversity of sub-communities inside.

The *Louvain algorithm* for community detection dynamically fragments the territory, showing its spatial heterogeneity across different scales. Thus, to properly understand the complex reality, we must first understand the spatial context where the algorithm is applied. For instance, the human interactions captured by the Twitter dataset transcend the traditional administrative boundaries. Zooming into multiple scales allows us to understand much better such interactions, and their effect on the markets, commercial agreements, and business opportunities, or even to avoid conflicts. The scalable structure of communities was recently used for implementing adaptive responses to COVID-19 restrictions in the United States. Buchel et al. ^37^ proposed to consider *multiscale social bubbles* for lifting shelter-at-home and mobility restrictions. Dwellers created social bubbles to minimize infection rates locally, while the different US states proposed travel zones to minimize transmissibility between remote areas. The analysis of mobility patterns has contributed to define the limits of human interactions and to assess the effects of the policies adopted by authorities, providing valuable information to policy-makers for adopting more effective travel restrictions, as well as quarantine policies that minimize the disruption of socio-economic activities.

### Spatial patterns

Some of the most influential factors behind the complexity of the social space are related to household income. From a social and behavioral perspective, income determines our lifestyle and world perception. Eagle et al.^38^ demonstrated that wealthy people travel more frequently and to more places. There is also a positive payoff in some cities between commuting farther for better jobs, while keeping better housing conditions^39,40^. Other studies analyze the correlation between social diversity and economic prosperity. Yong^41^ showed how the wealthiest regions develop much more complex and heterogeneous social networks where the emergence of labor opportunities can occur more easily.

Depending on the spatial scale and level of data aggregation, income composition allows to differentiate between an urban world, increasingly dynamic and wealthy, and a rural world in crisis. However, at local scales, we can also observe how some well-known urban regions are relatively poor, and some rural regions are relatively wealthy. In this way, the spatial scale is very relevant for properly understanding the complexity behind income-related human dynamics.

In the last few decades, the ideological and political division between rural and urban regions has escalated in the United States and other western countries^42,43^. Many policy experts attribute the spread of reactionary movements against globalization to the increasing confrontation between rural and urban voters. Just a few years ago, Brexit or the Trump victory in the 2016 US Presidential election were the most notable examples of these reactionary movements. Traditional division between American voters shows an evident spatial pattern that is always mentioned in media: while the Democratic Party concentrates most of its votes in the urban regions in the two coastlines, the Republican Party is the most voted in the central states. However, this spatial pattern is more complex than a simple division between the rural and urban America, especially in the face of a very polarized electoral scenario^44,45^.

Results from the 2016 US Presidential election showed that rural people accounted for only about 15% of the national population. Although rural voters preferred Trump and they certainly contributed to Republicans’ victory, they were not enough to swing the election’s results on its own nor to support the media rhetoric of a *rural revolt*^46^. Instead, Trump combined rural and small city over-performance in the industrial midwest. In other words, Trump voters were not so rural. In fact, the majority of Trump voters came from suburban areas where dwellers commute to work in some medium or large city. However, this spatial pattern diverges depending on the context and other additional factors. For example, the Latino vote in Florida is different from other states in the US. This pattern is particularly explained by the importance of Latin American voters, some of whom are residents with medium–high incomes living in the most important cities. Politically, neither the *Blue America* is so blue, nor the *Red America* is so red in political terms.

**Figure 5:**
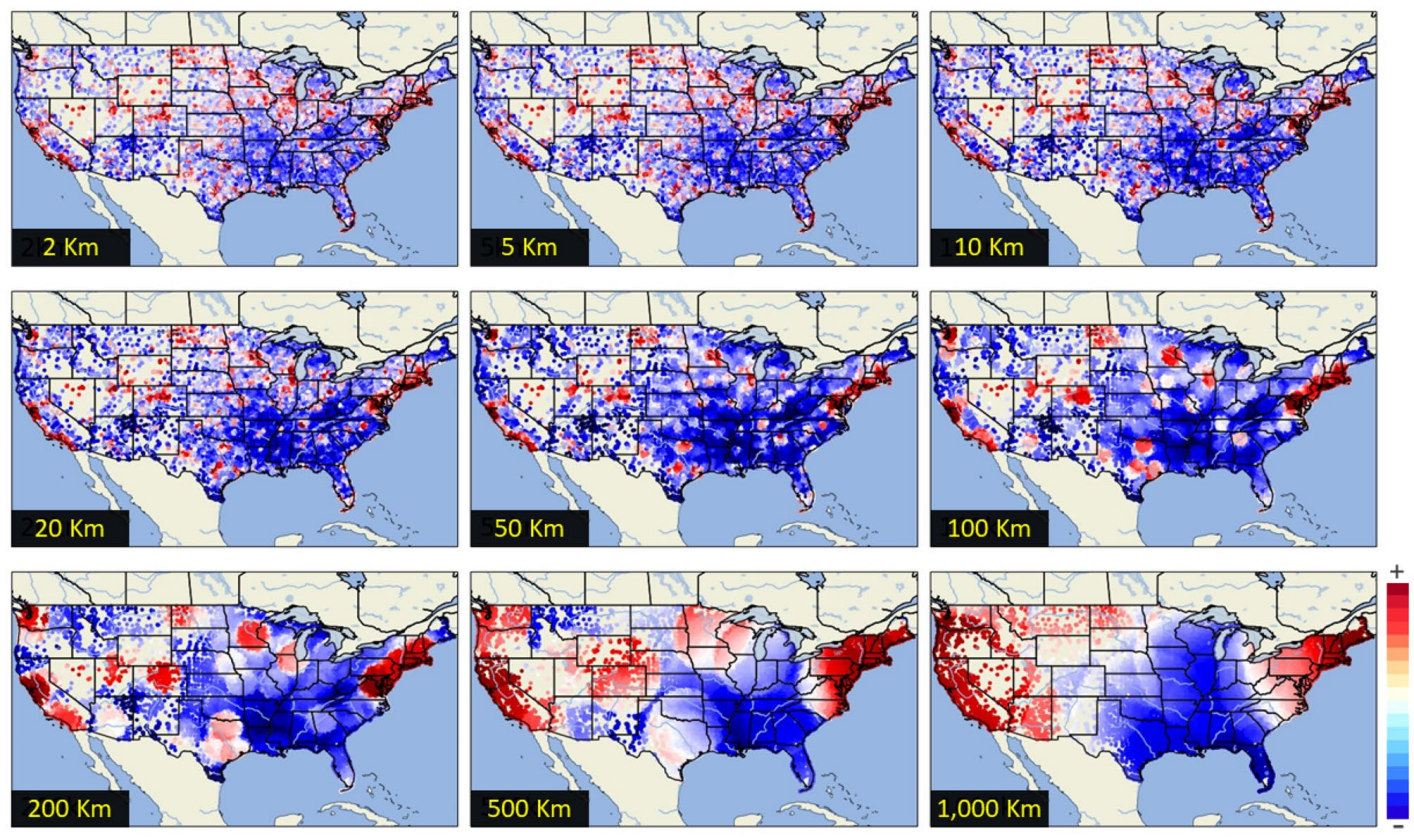
Income composition in the United States mainland by considering different aggregation levels—from 2 to 1000 km. Regions below national average income are shown in blue, while regions above national average income are shown in red. White-colored regions that emerge as gaps show regions with similar values to the national average. This figure was generated in Python using the library #Cartopy.

Figure 5 shows the income composition in the United States by considering the influence of the mesh size in data aggregation. Each individual node corresponds to a *census tract*, whose area is roughly equivalent to a neighborhood with 2500–8000 people. Data aggregation is conducted by applying a circular buffer whose radius ranges from 2 up to 1000 km. Mesh size considers all the nodes within the buffer, showing an interconnected effect all in all. The larger the buffer size, the higher the computational costs are.

Different income compositions emerge according to the level of data aggregation. With smaller buffers, a very granular pattern shows a high entropy and spatial diversity. As buffer size increases, complete cities emerge as wealthy areas in contrast to poorer and extensive rural areas showing an evident polarization of urban versus rural regions. Significant differences in wealth between cities emerge at a aggregation distance of 100 km. Larger distances draw the Eastern and Western sectors as the only wealthy regions, whereas Central America is shown as a large economically deprived region.

**Figure 6:**
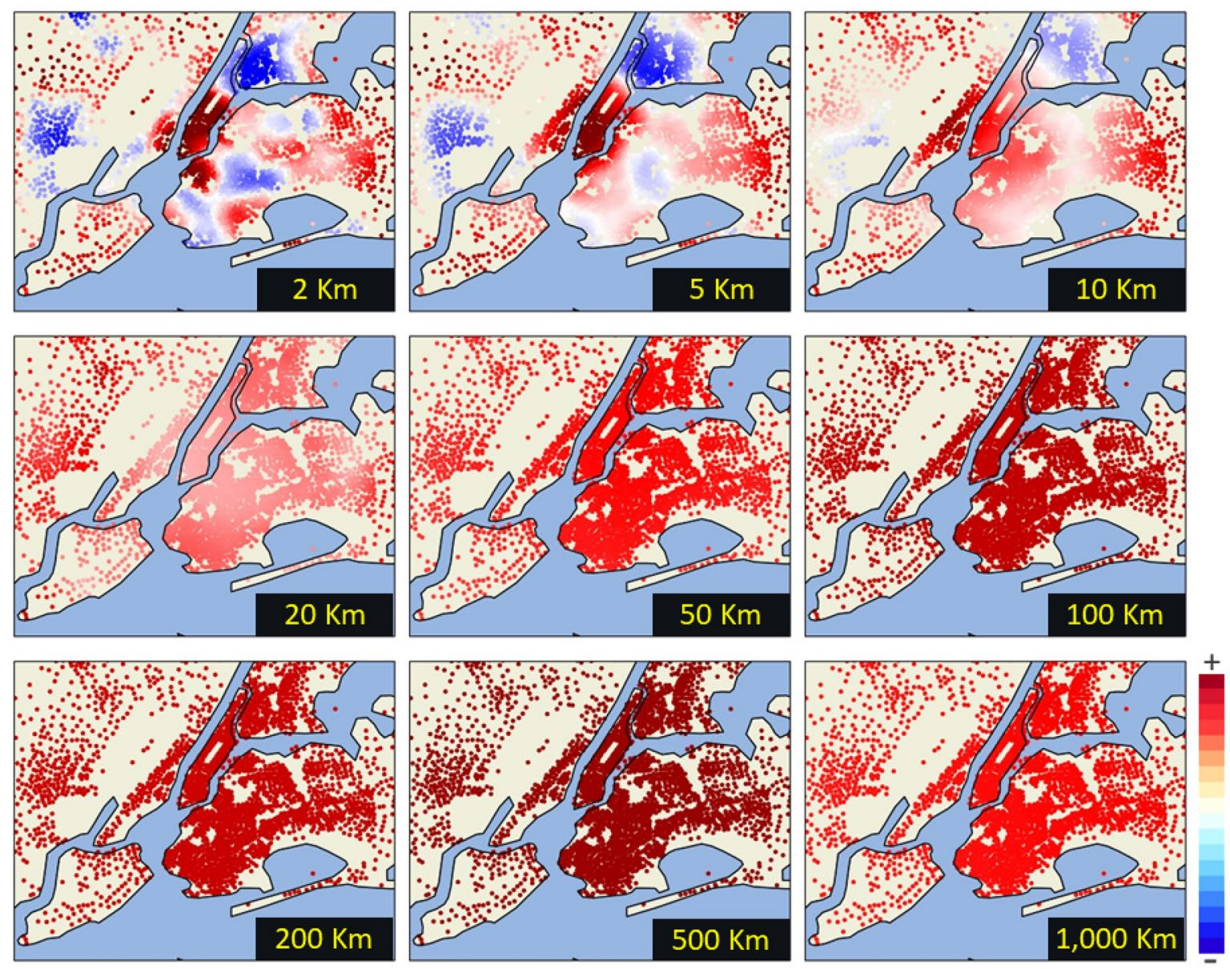
Income composition in New York City by considering different aggregation levels—from 2 to 1000 km. Regions below national average income are shown in blue, while regions above national average income are shown in red. White-colored regions that emerge as gaps show regions with similar values to the national average. This figure was generated in Python using the library #Cartopy.

On the other hand, zooming into New York City (Fig. 6) we can understand much better the income composition across intra-urban scales. At short distances, neighborhoods in blue have a low average income. However, these fade with buffers larger than 20 km showing the whole city as a wealthy region.

**Figure 7:**
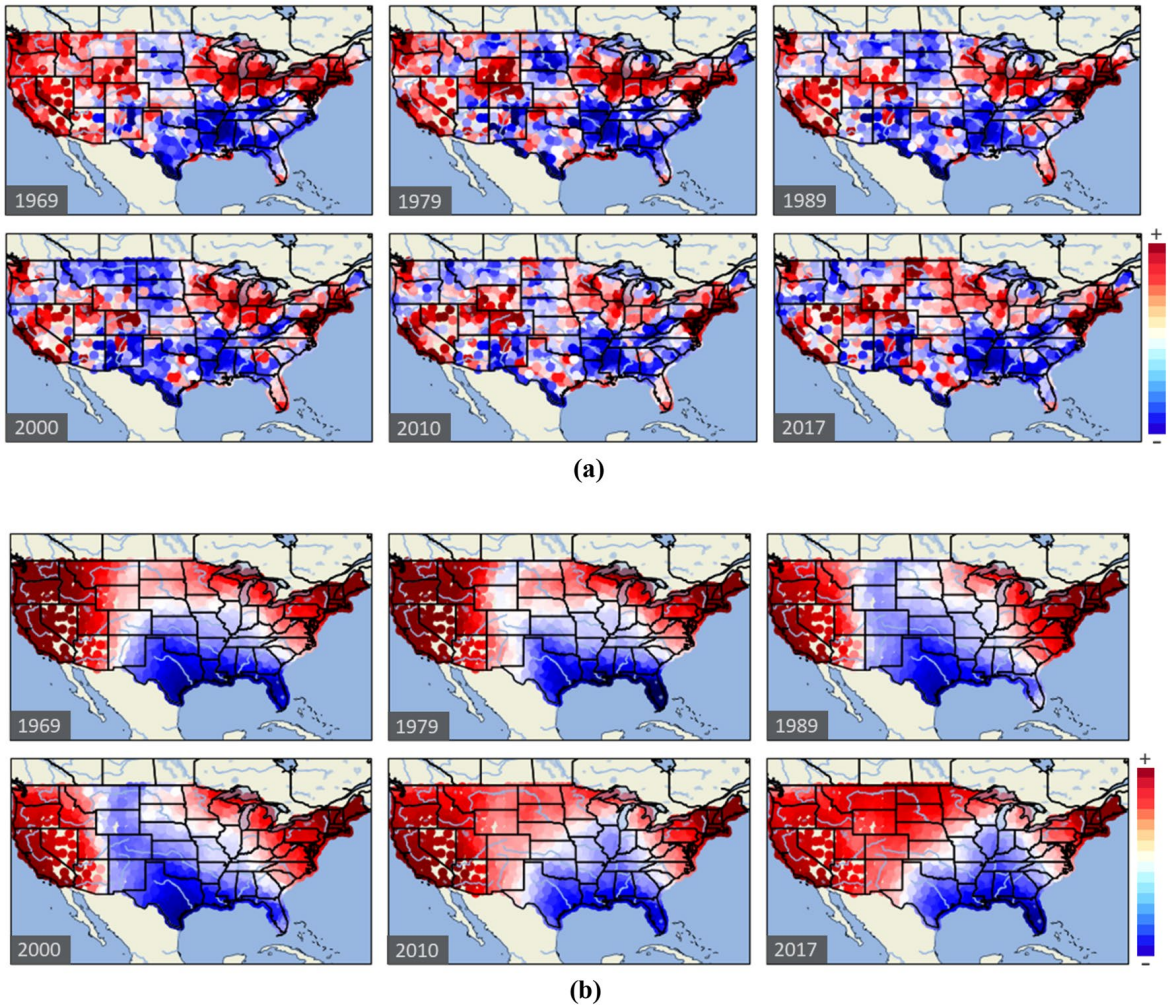
Income composition in the United States mainland over time by considering different aggregation levels: (**a**) 100 km and (**b**) 1000 km. Six years are represented: 1969, 1979, 1989, 2000, 2010, and 2017. Regions below national average income are shown in blue, while regions above national average income are shown in red. White-colored regions that emerge as gaps show regions with similar values to the national average. This figure was generated in Python using the library #Cartopy.

A similar approach is conducted for analyzing income composition across the United States over time. Figure 7 shows the evolution from the year 1969 to 2017 considering six individual years and two unique aggregation levels: 100 and 1000 km. The methodology used is the same as applied before, but instead of census tracts, we estimate spatial patterns using *counties* due to data limitations. This exercise enables us to validate the previous results obtained with the census tracts, but also to substantively reduce computational costs due to the lower number of nodes.

At an aggregation distance of 100 km, we can observe the high spatial diversity between rich and poor regions. The wealth concentration is mostly dominated by the metropolitan areas showing the division between rural and urban regions. Just a few cities concentrate most of the national wealth^47^. In general, we can observe how poverty and wealth present a consistent structure over time. The poorest regions located in the southeastern sector remain poor, whereas the wealthiest regions located in the northeast coast corridor and California coastline remain wealthy over time. However, the boundary between poverty and wealth has been shifting over time. In particular, certain regions located in the central states have fluctuated between wealth and poverty over time. Additionally, some cities have collapsed at some point, leading to an impoverishment of the surrounding regions due to their high dependence on those cities. In network science, this demonstrates the high collapse risks in hyper-connected systems motivated by cascading effects. This is particularly significant in the Detroit region, which was wealthy in the past, but it became increasingly poor in recent years.

At larger aggregation distances, the income composition is enormously simplified showing 2–3 vast regions whose borders have shifted over time. Wealth is mostly concentrated on the East and West coastlines, whereas the central region is mostly distressed. In the most recent decades, industrial relocation processes and the strong attraction of the most populated cities explain the decline of vast inland regions.

## Discussion and conclusions

Humans attempt to reduce the real-world complexity to make it more manageable^48^. Simplification helps to address all those complex concepts, but does not provide the full picture. In social sciences, many scholars analyze human dynamics and social behaviors by considering very specific scales and contexts. Moreover, many studies obviate the complexity and spatial heterogeneity of these dynamics and behaviors without applying a holistic perspective, being particularly evident in a networked society^49^. Addressing these aspects in social sciences is crucial, especially in the age of *information overload* where our instinct for categorization often leads us to an abstraction, encouraging polarization, rigid thinking, and outright denialism^50^*.*

From an economic perspective, countries can be rich and poor according to some indicators such as the GDP per capita, the level of economic development, and the natural resources they have. However, many poor people live in rich countries, and many rich people live in poor countries. This same dynamic is shown across different spatial scales ranging from the entire world to the smallest neighborhood. Spatial entropy based on socioeconomic factors shows how relatively rich citizens may live in relatively poor neighborhoods, and vice versa. Something similar occurs with the economic preferences of an immigrant that makes decisions based on job opportunities and living costs; it may be just more optimal for someone to work in a relatively less wealthy city with lower associated living costs than in a richer city with very high associated living costs. Therefore, the social space is physically partitioned showing different spatial patterns across multiple scales.

In this paper, we have illustrated the complexity of the social space as a multiscale function using data related to human interactions and income composition. We aggregated data across multiple scales showing substantial changes in spatial patterns. Results related to community detection range from clustering supranational communities to regional/local communities, while income composition ranges from coastal-inland divergence to urban segregation patterns in the United States.

Social systems operate across multiple scales. Individuals associate in groups, organizations, communities, and geographical regions^51^. This information is part of the self-organizing process of social systems and it can be used to improve the quality of explanatory and predictive models, as well as to enrich interpretations. The success of social systems depends on their capacity to achieve effective collective actions and to respond to the demands of social behaviors^52^. However, the multiscale operability of social systems is not fully understood yet. Some statistical assumptions related to normality do not always hold, and the blind application of black-box algorithms for data analysis could lead to inconsistent and biased results. Social scientists must consider the existence of fractal dimensions and problems derived from MAUP in mapping, but also the high spatial heterogeneity of human behavior, which must be framed in its right geographical context. This concern must be better emphasized in particular with the increasing importance of the *Artificial Intelligence*, as some experts point to the need to develop an *Anthropological Intelligence* with a sense of social context^53^.

We have also shown the relevance of adopting systemic perspectives on the social space, introducing concepts and approaches from complex systems. It may help to build a more complete picture of the social space by incorporating variables such as the context and the interactions among agents. Finally, results contribute to strengthening the discussion on this issue, which could be valuable for policy-makers to adopt the most optimal decisions for the benefit of society. Future studies will delve further into the concepts presented in order to better understand the complexity of the social space.

## Data Availability

The datasets used and/or analyzed during the current study available from the corresponding author on reasonable request.
